# Weight-Loss Strategies Used by the General Population: How Are They Perceived?

**DOI:** 10.1371/journal.pone.0097834

**Published:** 2014-05-22

**Authors:** Chantal Julia, Sandrine Péneau, Valentina A. Andreeva, Caroline Méjean, Léopold Fezeu, Pilar Galan, Serge Hercberg

**Affiliations:** 1 Paris 13 University, Sorbonne Paris Cité, Nutritional Epidemiology Research Team, Epidemiology and biostatistics Center, Cnam, Paris 5 University, Paris 7 University, Bobigny, France; 2 Department of Public Health, Avicenne Hospital, Bobigny, France; Oklahoma State University, United States of America

## Abstract

**Background:**

The rising prevalence of obesity and the social pressure for thinness increase the prevalence of dieting. However, little is known about the overall perception of dieting strategies actually used by the general population.

**Objectives:**

Our main objective was to investigate perceptions of weight-loss practices in an observational study in order to identify the most favourable strategy.

**Design:**

Adults from the ongoing Nutrinet-Santé cohort study who had reported engaging in dieting in the three previous years were included in the study. For each diet, detailed information was collected on types of diets, circumstances and perception of the diet, and outcomes. Perceptions were compared across diets using sex-specific mixed effects models.

**Result:**

Among the 48 435 subjects who had completed the respective questionnaire, 12 673 (26.7%, 87.8% of women) had followed at least one weight-loss diet in the previous three years. Diet plans prescribed by health professionals and diets conforming to official dietary recommendations were the most favourably perceived among all assessed weight-loss strategies. Alternatively, commercial diet plans and self-imposed dietary restrictions were more negatively perceived (Odds ratios (OR) for adherence difficulty 1.30 (95% confidence interval (0.99;1.7)) in men and OR 1.92 (1.76;2.10) in women compared to official nutritional guidelines; OR 1.06 (0.82;1.38) in men and OR 1.39 (1.26;1.54) in women respectively) compared to official nutritional guidelines.

**Conclusion:**

Official dietary recommendations could be useful tools for maintaining a dietary balance while following a weight-loss diet.

## Introduction

Prevalence of obesity in industrialized countries has been continuously rising for more than three decades.[1], [2] Elevated disease risks linked to overweight and obesity have led authorities to consider this epidemic a major public health challenge.[3], [4] Moreover, social pressure to adhere to a thin body image ideal exposes those not meeting it to consequences encompassing both personal and professional challenges.[5], [6] Both medical concerns and this social pressure for thinness drive more and more people to dieting.[7]–[9] In the USA in 2005–2006, 36.9% of men and 57% of women had been on some type of a weight loss diet in the year preceding the report, which represented a 10% increase for women compared to 5 years earlier.[9], [10]

In this context, the weight-loss market has been growing rapidly in response to the demand and now includes a number of commercial diet plans/programs, self-help books written by “celebrity-status” physicians, mobile diet applications and websites, and word-of-mouth marketing. In 2011, the weight-loss market was estimated to be worth $60 billion in the USA[11], and almost 3 billion € in France.[12] Very few commercial diet plans have accepted to be evaluated using scientific rationale and methodology[13]–[15], instead relying on anecdotal evidence to establish their effectiveness.[16] Aside from well-defined commercial offers or medical advice, individuals can also self-impose dietary restrictions.[17] In addition, official dietary recommendations and guidelines issued by the respective authorities can serve as alternate and reliable sources of information for individuals wishing to achieve a balanced diet in order to lose weight.[18], [19] Finally, in specific clinical settings, nutritional medical counselling is recommended.

In spite of the large number and diversity of the available weight-loss strategies, little is known about their actual use in the population or the perception of each strategy by the consumers.[16]

Irrespective of the type of weight-loss diet used, weight regain is common, often reaching or even exceeding the initial weight.[20]–[22] A major determinant of weight loss and weight maintenance is adherence to the diet,[23] which is dependent on the perception of and the conditions under which the diet is followed.[24], [25] However, the literature on these aspects of weight-loss practices is scarce. To our knowledge, no study has yet described in detail the various weight-loss strategies in a large sample of the general population, or has documented perceptions of weight-loss diets.

Our objectives were to investigate perceptions of weight-loss strategies in a large sample of the general population in order to identify the best perceived strategy. We hypothesized that weight-loss strategies would differ by gender, and we therefore conducted separate analyses for men and women.[9], [26], [27]

## Materials and Methods

### Population

Participants were selected among subjects included in the Nutrinet-Santé cohort, a prospective observational study with a scheduled follow-up of 10 years in which inclusion and follow-up are performed on the Internet using a dedicated and secure website. The main objectives of the Nutrinet-Santé study are:1) to investigate the relationships between nutrition and health; and 2) to elucidate the role of various determinants of dietary patterns and nutritional status, and their interactions. Inclusion in the study began in May 2009 and is still ongoing.

Detailed information about the design/methods and rationale of the Nutrinet-Santé study can be found elsewhere.[28]

Briefly, volunteers from the general population aged >18 years are considered actively enrolled in the cohort upon completion of a set of online self-administered questionnaires assessing diet (via three dietary records randomly distributed over a two-week period), physical activity, anthropometrics, lifestyle and socio-economic conditions and health status. Once the subjects are included in the study, they receive additional online self-administered questionnaires on a monthly basis, on specific topics pertaining to dietary behaviours, determinants of eating habits and health status. Between April 1^st^, 2012 and October, 2012, all included subjects received a follow-up questionnaire pertaining to weight loss strategies, with a sub-part of it pertaining to perceptions of weight-loss strategies (up to four) used in the last three years. Self-reported weight and height were asked for in the same questionnaire. All subjects having completed this questionnaire and having followed at least one weight-loss diet over that period were eligible for the present analyses.

### Ethics statement

The study is conducted in accordance with the Declaration of Helsinki, and all procedures have been approved by the International Research Board of the French Institute for Health and Medical Research (0000388FWA00005831) and the *Commission Nationale Informatique et Libertés* (908450 and 909216). Electronic informed consent was obtained from all participants.

### Data collection

#### Diets' circumstances and perception

Dieting circumstances and perceptions were assessed through a questionnaire administered to all participants between April 1^st^, 2012 and October, 2012.

All popular commercial diet plans were mentioned in the questionnaire so that subjects could readily identify the type of diet they had followed (e.g. Atkins, Dukan, Cohen, Weight Watchers, etc., see **Table S1**). Additional categories were included corresponding to self-defined dietary restrictions: ‘reducing snacking’, ‘reducing fat intake,’ ‘reducing starchy foods,’, ‘reducing simple carbohydrates’ and combinations of these options. Other categories corresponded to the dietary recommendations disseminated in the general French population (including improving variety, limiting fats and added sugar intake, avoiding snacking, etc.) and to diets prescribed by a health professional.[18], [29]

The types of diet were grouped in the following five categories: 1)commercial weight-loss diet plans (including Atkins, Dukan, South beach, etc.); 2) commercial plans including coaching methods (eg, Weight Watchers, Jenny Craig etc.); 3) self-imposed dietary restrictions; 4) adherence to official dietary guidelines[18], [29]; and 5) diet prescribed by a health professional. Diets referred to in plain text were also recoded in the previous categories whenever possible.

For each diet declared, circumstances and perceptions of the diet were detailed. Circumstances of dieting pertained to diet duration, interval between diet cessation and questionnaire completion and concomitant physical activity. Attitudes and perceptions pertained to main reasons for diet cessation (including the following: ‘I attained my objective in terms of weight loss’, ‘I attained my objective in terms of duration’, ‘I stopped early because of health problems’, ‘I stopped early because it was too expensive’, ‘I stopped early because it was too complicated to follow’, ‘I stopped early because I felt frustrated or hungry’, ‘I stopped early because the diet was inefficient’), adherence difficulty (measured on a Likert scale including the following options: ‘Very easy’, ‘Quite easy’, ‘Moderately difficult’, ‘Difficult’ and ‘Very difficult’), complications experienced in everyday life, frustration experienced regarding food choices (measured on a Likert scales including the following options: ‘Not at all’, ‘A little’, ‘Moderately’, ‘A lot’, ‘Enormously’).

The questionnaire on diets was completed with questions on current weight and height.

#### Covariates

Age, educational level (highest diploma obtained), smoking status and occupational category were obtained through a self-administered questionnaire at inclusion in the study. Weight (in kg) and height (in cm) were obtained through the self-administered questionnaires on dieting practices previously described. Body mass index (BMI) from the diet questionnaire was computed as weight (in kg) divided by the square of height (in m).

### Statistical analyses

Outcome variables of interest were circumstances of the diet, perception during the diet and perception after diet cessation. The initial response scales were dichotomized given the number of responses in each category (eg, grouping together ‘Not at all’ and ‘A little’). Dichotomization of Likert scale responses was regarded as necessary given the distribution across response categories, and in order to maintain interpretation of results as simple as possible. Regarding diet cessation, the items corresponding to an achieved objective were grouped together, all other responses were considered as reasons for an ‘Early discontinuation of the diet’.

Characteristics of dieters and of the diets followed over the previous three years according to gender were compared using chi-square tests. Perceptions according to the type of diet were investigated separately for men and women, in univariable (using chi-square tests) and multivariable analyses. In order to take into account the hierarchical structure of data (diets within individuals) in the multivariable analyses, dichotomized perception variables were compared according to type of diet using multivariable marginal logistic mixed effects models, using the GENMOD procedure in SAS. Perception variables were introduced as dependent variables and types of diet as the independent variables in the models (reference category  =  adherence to official dietary guidelines). Results are presented as Odd Ratios (OR) and 95% confidence intervals (95%CI). The models were adjusted for factors pertaining to the diet (eg, total number of diets followed in the last three years (continuous), rank of the diet (categorical) – when more than one diet had been followed, interval between diet and questionnaire completion (categorical; <6months, [6–12 months[, [12–24 months[, ≥24 months)) and factors pertaining to the subjects (age (continuous), educational level (categorical; no diploma and primary, secondary, university), smoking status (categorical; never smoker, former smoker, current smoker), current BMI (continuous)). As some subjects had missing data on covariates pertaining to diets (namely interval between diet and questionnaires), mixed effect models were conducted on smaller samples.

We also investigated a potential effect modification of perceptions by number of achieved diets (single dieters vs. multiple dieters).

## Results

Between April 1, 2012 and October 12, 2012, 48 435 subjects had completed the questionnaire about dieting strategies. Among them, 12 628 subjects (26.7%) had followed at least one weight-loss diet in the previous three years (detailed information in the **Figure S1**). Compared to non-dieters having completed the questionnaire, dieters were more likely to be women (P<0.001), to be younger (P<0.001 for both men and women), to be currently employed (P<0.001 for both men and women) and to be heavier (P<0.001) and to be former smokers (P<0.001 for both men and women) (see **Table S4**).

Compared to female dieters, male dieters tended to be older, more frequently former smokers, more frequently overweight or obese, and less likely to have followed more than one diet in the previous three years. (**Table 1**) Diet characteristics were also significantly different across genders: compared to female dieters, male dieters were less likely to select commercial diet plans (with or without coaching programs), and more likely to undergo self-imposed dietary restrictions (**Table 2**). Among the commercial diet plans, the Dukan diet was the most frequently cited in both men and women (**Table S2**). As to commercial diet coaching programs, Weight Watcher was the most represented (**Table S2**).

**Table 1:** Characteristics of dieters included in the study.

		Men		Women		
*N*		*1544*	*12.2*	*11084*	*87.8*	
		N	%	N	%	P
Age	*≤25 y-o*	26	1.7	503	4.5	<.0001
	*]25*–*40] y-o*	356	23.1	3671	33.1	
	*]40*–*60] y-o*	624	40.4	4982	45	
	*>60 y-o*	538	34.8	1928	17.4	
Educational level	*No diploma and primary*	292	18.9	1780	16.1	0.01
	*Secondary*	295	19.1	2305	20.8	
	*University*	957	62.0	6999	63.2	
Current employement status	*Work*	966	62.6	7314	66.0	0.008
	*Inactive*	578	34	3770	34.0	
Smoking status	*Never smoker*	558	36.1	5302	47.8	<.0001
	*Former smoker*	765	49.6	4150	37.5	
	*Current smoker*	221	14.3	1632	14.7	
BMI	*<25 Normal weight*	505	32.7	6308	56.9	<.0001
	*[25*–*30[ Overweight*	734	47.5	3207	28.9	
	*≥30 Obese*	305	19.8	1569	14.2	
Number of diets in the previous three years	*1*	1027	66.5	6493	58.6	<.0001
	*>1*	517	33.5	4591	41.4	

**Table 2:** Characteristics of the diets performed.

		Men (n = 2311)		Women (n = 18036)		
		N	%	N	%	P^a^
Type of diet	*Adherence to dietary recommendations*	721	31.2	5311	29.5	<.0001
	*Commercial diet plan*	703	30.4	6152	34.1	
	*Commercial coaching program*	75	3.3	2280	12.6	
	*Self-imposed dietary restrictions*	759	32.8	3867	21.4	
	*Prescribed by a health professional*	53	2.3	426	2.4	
**Conditions of the diet**						
Time from diet	*<6 months*	319	14.2	2411	13.9	0.08
	*[6*–*12[ months*	315	14.0	2615	15.0	
	*[12*–*24[ months*	657	29.3	5405	31.1	
	*≥24 months*	955	42.5	6958	40.0	
Reason for diet cessation	*Objective attained*	1382	59.8	8186	45.4	<.0001
	*Early stop to the diet*	929	40.2	9846	54.6	
Diet duration	*<1 month*	1373	59.4	11287	62.6	0.003
	*≥1 month*	938	40.6	6746	37.4	
Physical activity associated to the diet	*Yes*	1099	47.6	7999	44.4	0.003
	*No*	1212	52.4	10034	55.6	
**Perception of the diet**						
Adherence difficulty	*Easy to follow*	1551	67.1	11161	61.9	<.0001
	*Difficult to follow*	760	32.9	6873	38.1	
Experiencing complications	*Not all to a little*	1755	75.9	12224	67.8	<.0001
	*Moderately to enormously*	556	24.1	5810	32.2	
Experiencing frustration	*Not all to a little*	705	30.5	4866	27.0	<.0001
	*Moderately to enormously*	1606	69.5	13168	73.0	
Hunger during dieting	*Not at all*	1064	46.0	8426	46.7	0.54
	*A little to enormously*	1247	54.0	9608	53.3	

Comparison between men and women.

a P value obtained with chi-square tests.

For all other analyses, results of cross-tabulations (comparison across diet types with Chi-square tests) are presented in **Tables S3 and S4**. Results of multivariate analyses are presented in **Tables 3** **and** **4**.

**Table 3:** Diet perception according to type of diet performed – Multivariable analysis – Men.

	Adherence to dietary recommendations	Commercial diet plan			Self-imposed dietary restrictions			Commercial coaching programs			Diet prescribed by a health professional			P
	OR	OR	95%IC	P	OR	95%IC	P	OR	95%IC	P	OR	95%IC	P	
***Conditions of the diet***														
Early stop to the diet	**1**	**0.67**	(0.53;0.85)	0.001	**1.03**	(0.82;1.30)	0.782	**1.24**	(0.83;1.88)	0.297	**0.84**	(0.46;1.54)	0.566	0.0006
Diet duration >1month	**1**	**0.53**	(0.41;0.69)	<.0001	**0.74**	(0.57;0.97)	0.031	**0.90**	(0.46;1.75)	0.756	**1.53**	(0.70;3.30)	0.290	<.0001
Concomitant physical activity	**1**	**0.56**	(0.45;0.71)	<.0001	**0.75**	(0.60;0.94)	0.014	**0.59**	(0.34;1.02)	0.058	**1.07**	(0.57;2.01)	0.835	<.0001
***Perception of the diet***														
Adherence difficulty	**1**	**1.30**	(0.99;1.7)	0.051	**1.06**	(0.82;1.38)	0.659	**0.91**	(0.48;1.74)	0.781	**1.40**	(0.70;2.82)	0.341	0.313
Experiencing complications	**1**	**2.32**	(1.77;3.07)	<.0001	**0.87**	(0.65;1.17)	0.358	**1.84**	(0.95;3.59)	0.072	**1.81**	(0.84;3.06)	0.119	<.0001
Experiencing frustration	**1**	**1.18**	(0.91;1.54)	0.206	**0.76**	(0.60;0.97)	0.027	**0.56**	(0.32;0.99)	0.044	**0.59**	(0.29;1.20)	0.146	0.004
Hunger during dieting	**1**	**0.81**	(0.64;1.02)	0.078	**1.23**	(0.99;1.53)	0.063	**0.54**	(0.30;0.95)	0.033	**0.78**	(0.41;1.50)	0.454	0.001

The models were adjusted for factors pertaining to the diet (eg, total number of diets followed in the last three years (continuous), rank of the diet (categorical) – when more than one diet had been followed, interval between diet and questionnaire completion (categorical; <6months, [6–12 months[, [12–24 months[, ≥24 months)) and factors pertaining to the subjects (age (continuous), educational level (categorical; no diploma and primary, secondary, university), smoking status (categorical; never smoker, former smoker, current smoker), current BMI (continuous)).

**Table 4:** Diet perception according to type of diet performed – Multivariable analysis – Women.

	Adherence to dietary recommendations	Commercial diet plan			Self-imposed dietary restrictions			Commercial coaching methods			Diet prescribed by a health professional			P
	OR	OR	95%IC	P	OR	95%IC	P	OR	95%IC	P	OR	95%IC	P	
***Conditions of the diet***														
Early stop to the diet	**1**	**0.95**	(0.87;1.03)	0.216	**1.14**	(1.03;1.26)	0.009	**1.01**	(0.90;1.13)	0.875	**0.93**	(0.73;1.19)	0.58	0.007
Diet duration >1month	**1**	**0.56**	(0.51;0.61)	<.0001	**0.75**	(0.68;0.83)	<.0001	**1.29**	(1.15;1.44)	<.0001	**1.75**	(1.37;2.24)	<.0001	<.0001
Concomitant physical activity	**1**	**0.57**	(0.53;0.62)	<.0001	**0.75**	(0.69;0.82)	<.0001	**0.95**	(0.86;1.06)	0.358	**0.93**	(0.76;1.14)	0.505	<.0001
*Perception of the diet*														
**Adherence difficulty**	**1**	**1.92**	(1.76;2.10)	<.0001	**1.39**	(1.26;1.54)	<.0001	**0.70**	(0.62;0.80)	<.0001	**0.88**	(0.69;1.12)	0.302	<.0001
Experiencing complications	**1**	**2.43**	(2.22;2.67)	<.0001	**1.03**	(0.92;1.15)	0.652	**1.27**	(1.12;1.43)	0.0001	**1.03**	(0.81;1.31)	0.800	<.0001
Experiencing frustration	**1**	**1.57**	(1.42;1.73)	<.0001	**1.08**	(0.98;1.20)	0.136	**0.48**	(0.43;0.54)	<.0001	**0.75**	(0.59;0.94)	0.013	<.0001
Hunger during dieting	**1**	**0.83**	(0.76;0.90)	<.0001	**1.26**	(1.14;1.38)	<.0001	**0.69**	(0.62;0.77)	<.0001	**0.64**	(0.52;0.80)	0.0001	<.0001

The models were adjusted for factors pertaining to the diet (eg, total number of diets followed in the last three years (continuous), rank of the diet (categorical) – when more than one diet had been followed, interval between diet and questionnaire completion (categorical; <6months, [6–12 months[, [12–24 months[, ≥24 months)) and factors pertaining to the subjects (age (continuous), educational level (categorical; no diploma and primary, secondary, university), smoking status (categorical; never smoker, former smoker, current smoker), current BMI (continuous)).

### Conditions of the diet

#### Reasons for diet cessation

Overall, 40.2% of men and 54.6% of women (P<0.001) reported reasons for diet cessation corresponding to early discontinuation (**Table 2**). The commercial coaching programs were those leading to the largest proportion of early cessations (**Table 3** **and** **4**).

#### Duration of the diet

Overall, 40.6% of men and 37.4% of women followed a diet for more than a month (P = 0.003) (**Table 2**). Commercial diet plans and self-imposed dietary restrictions tended to be followed for shorter periods of time compared to adherence to official dietary guidelines: commercial diets were less likely to be followed for more than a month (OR = 0.53; CI: 0.41;0.69 in men; OR = 0.56, CI: 0.51;0.61 in women), whereas diets prescribed by health professionals were more likely to be followed for more than a month (OR = 1.53, CI: 0.70;3.30 in men, OR = 1.75, CI: 1.37;2.24 in women) compared to adherence to official dietary recommendations (**Table 3** **and** **4**).

#### Physical activity concomitant with the diet

Overall, 47.6% of men and 44.4% of women reported concomitant physical activity when dieting (P = 0.003). However, subjects on commercial diet plans were less likely to include a physical activity component while dieting compared to those adhering to official dietary guidelines (**Table 3** **and** **4**).

### Perceptions of the diet

#### Adherence difficulty

Overall, 32.9% of men and 38.1% of women perceived the diet they had followed as not easy to follow. Compared to adherence to official dietary guidelines, commercial diets were perceived as less easy to follow (OR = 1.30, CI: 0.99;1.70 in men, OR = 1.39, CI: 1.26;1.54 in women) and commercial coaching programs as being easier to follow (OR = 0.91, CI: 0.48;1.74 in men; OR = 0.70, CI: 0.62;0.80 in women).(**Tables 3** **and** **4**)

#### Complications experienced in everyday life

Overall, 24.1% of men and 32.2% of women considered the diet they had followed as creating complications in their everyday life. Compared to adherence to official dietary guidelines, commercial diets and commercial diet coaching programs were perceived as more complicated (OR = 2.32, CI: 1.77;3.07 and 1.84, CI: 0.95;3.59, respectively, in men; OR = 2.43 CI: 2.22;2.67 and 1.27, CI: 1.12;1.43, respectively, in women) (**Table 3** **and** **4**).

#### Frustration experienced regarding food choices

Overall, 69.5% of men and 73% of women reported having experienced frustration regarding food choices while dieting. Subjects following commercial coaching programs and diets prescribed by health professionals reported significantly less frustration than those adhering to dietary recommendations (OR = 0.56, CI: 0.32;0.99 and 0.59, CI: 0.29;1.20, respectively in men; OR = 0.48, CI: 0.43;0.54, and 0.75, CI: 0.59;0.94, respectively, in women) (**Table 3** **and** **4**).

#### Hunger during dieting

Overall, 54.0% of men and 53.3% of women reported feeling hungry when dieting (P  = 0.54). Self-imposed dietary restrictions were significantly associated with more hunger compared to adherence to dietary guidelines (**Table 3** **and** **4**).

Overall, number of achieved diets were not an effect modifier of diet perception by subjects, single dieters rating the same diet the same way as multiple dieters.

## Discussion

The prevalence of dieters in our sample is comparable to that reported in a representative sample of the French population.[30] However, compared to other international and particularly North American studies, the prevalence of weight-loss strategies in our population was relatively low.[9], [10]This can be related to the fact that dieting behaviors are closely related to BMI, and compared to the rates in the USA, France has a lower prevalence of overweight/obesity.[31], [32] Still, our interest was not characterization of dieters per se, or giving population-level estimates of dieting outcomes.

As hypothesized, circumstances and types of diets followed by men or women differed markedly. Gender differences in the prevalence of dieting have been consistently reported[9], [26], [27] and can be explained by double standards regarding the social pressure for thinness, driving body dissatisfaction.[33] Women start experiencing social (and even family) pressure for thinness and body dissatisfaction at a very early age,[34], [35] and therefore start dieting earlier and more repeatedly.

Consistent with our results, results from NHANES showed that strategies for losing weight differed according to gender: men were found to be more likely to exercise than were women and less likely to join a weight-loss program.[9], [27] Differing strategies for losing weight according to gender can be related to differing weight goals when dieting, with women tending to have higher weight loss goals than men.[27], [36] Our results extend these findings, showing that despite differences in the choices made by men and women in terms of diet strategies, their perceptions tend to be similar.

Diets prescribed by health professionals were associated with the most positive perceptions: they were perceived as being relatively easy to follow, as leading to reduced frustration, and more importantly, as being less likely to lead to weight regain or to dietary imbalance following cessation. Health professionals are likely to be aware of the importance of setting achievable goals for dieters[37], and of the health impact of even modest weight loss.[21] In fact, expert recommendations now tend to shift from low- and very-low-calorie diets to moderate restrictions[38], [39] concomitant with physical activity[40] in order to achieve progressive weight loss. Finally, health professionals personalize dietary advice to individual lifestyles and dietary preferences. They are often engaged in the individualized and active follow-up of their patients. All of these considerations could explain the positive perceptions that such diets generate.

On the other hand, commercial diet plans and self-imposed dietary restrictions appear to entail the least favorable perceptions. Popular commercial diets rely mostly on calorie restrictions regarding specific macronutrients and are often quite different from dietary recommendations.[41] The Dukan and Atkins diets, for example, are low-calorie diets with very low carbohydrate and very high protein intakes.[42] Thus, alterations in macronutrient supply can alter signals of hunger and satiety.[25], [43] Regardless of the macronutrient composition, the extent of calorie restriction has been negatively related to compliance with the diet.[44], [45] Such characteristics of commercial diet plans are likely to explain at least in part the negative outcomes we observed in terms of perceptions. Moreover, exercise concomitant with dietary restriction is also an important determinant of weight loss and weight-loss maintenance in the long term.[46] We observed that commercial diets were not likely to be followed in parallel with exercise. This result is consistent with those of a qualitative study in a sample of obese subjects indicating that whereas subjects engaged repeatedly in commercial diets, few engaged in physical activity.[47]

Following a generally balanced diet informed by official dietary recommendations appeared to be a well perceived strategy for losing weight, often equally favorably perceived as a diet prescribed by a health professional. In France, the Programme National Nutrition Santé is a national public health program aiming at improving through nutrition the health status of the general population.[29] Initially set in 2000 for a period of five years, it has regularly been renewed and is now in its third phase.[48] While public health objectives pertaining to the lowering of the prevalence of overweight and obesity are included in the program's framework, no specific dietary recommendations have been disseminated as to optimal weight.[29], [48] Here, we observed that these dietary recommendations have empowered subjects to balance their own diets in order to lose weight, leading to positive results. This could prompt authorities to enlarge current recommendations in order to encompass advice for weight maintenance and optimal weight. However, such dietary recommendations should be carefully developed in order not to increase the already substantial social pressure regarding thinness or the stigmatization of obese subjects.[49]

Among all dieting strategies, commercial coaching programs appeared to be midway between commercial diet plans and the adherence to recommendations as regards perceptions of the diet, feelings of hunger, frustration. Weight loss in commercial coaching programs is mostly related to retaining rate, with the most active subjects in the programs having the most beneficial results.[13], [50], [51] Participation in weight-loss programs is hindered by costs, as the most frequent motivation for dropping out of the program is its cost at the individual level.[52] This could explain in part our results, especially as regards subjects who were not able to complete the program for financial reasons even though they had perceived it positively.

### Strengths and limitations

Strengths of our study include the comprehensive investigation of dieting behaviors and dieting perception in a large sample issued from the general population, an original categorization of diets according to their type as marketed to the public, and a multivariable analyses taking into account extensive adjustment, both at the subject's and at the diet levels.

Our results are derived from observational data exclusively. Subjects therefore self-selected the types of diets that they undertook. Therefore, the observed associations could be related to this self-selection of subjects to different types of diet rather than stemming from diet differences in themselves. However, taking into account such confounding is arduous, as determinants of choice of types of diets by individuals are bound to be complex.

Our study is subject to limitations. First, we retrospectively investigated the perceptions of the diets. Recall bias would be of particular concern in cases where the perceived long-term effectiveness of the diet was low. Indeed, long-term weight regain is likely to hinder perception of diets performed several years before. However, we were able to minimize this bias, taking into account related covariables, such as total number of diets followed, rank of the diet (among multiple diets) and time since diet cessation. Second, we were not able to document actual weight loss for each diet, which could have strengthened and extended our results pertaining to weight regain. However, our primary objective was to document perceptions of the various types of diets.

Our population included French residents, which live in a specific food environment, including a culture of diversified food behaviour, and a nutritional prevention public health program of more than a decade long.[29], [53] Moreover, weight loss popular diets differ across countries and time. However, if food environments are different across countries, it has been shown that dietary behaviors, perception of overweight and weight-loss attempts are comparable across Western countries.[54], [55] Finally, our sample was drawn from subjects included in an e-cohort investigating specifically nutrition. As such, our population is likely to be more health-conscious and more aware of the relationships between nutrition and health. Moreover, Internet recruitment of subjects can be prone to specific selection biases. However, we were able to show that the Nutrinet-Santé study has been able to recruit participants of various profiles, therefore limiting such bias. [56] However, given these limitations perception of dieting strategies may differ from the general population, altering the generalization of our results.

## Conclusions

In conclusion, our results show that beside diets prescribed by health professionals, which should be followed only in individually-determined clinical situations, counseling relying on dietary recommendations as they are disseminated in the general population could serve as a useful and well perceived strategy to achieve both dietary balance and weight control. Attention should be drawn to commercial diet plans, as they are both negatively perceived and give relatively poor results.

## Supporting Information

Figure S1 Flow-chart of inclusions in the study.
(DOCX)

Table S1 Detailed information on the type of diet followed.
(DOCX)

Table S2 Diet perception according to the type of diet followed – Cross-tabulations– Men (n = 2311).
(DOCX)

Table S3 Diet perception according to the type of diet performed – Cross-tabulations- Women (n = 18036).
(DOCX)

Table S4 Comparison between subjects having followed at least one weight loss diet in the past three years and subjects not having followed any; Analyses stratified by gender.
(DOCX)
