# Supplementary material for: Weight-Loss Strategies Used by the General Population: How Are They Perceived?
Source: PLoS One. 2014 May 22;9(5):e97834. doi: 10.1371/journal.pone.0097834 (PMC4031181; doi:10.1371/journal.pone.0097834)
Supplement: Figure S1 — Flow-chart of inclusions in the study. (DOCX) [file pone.0097834.s001.docx]

34 842 subjects

Had not followed any weight loss diet in the previous three years

964 subjects

Had missing baseline socio-demographical data

13 474 subjects

Did not complete the diet questionnaire

12 628 subjects

Having followed at least one weight loss diet in the previous three years

47 470 subjects

Had complete socio-demographical and anthropometric baseline data

48 435 subjects

Completed the diet questionnaire

61 909 subjects

Received to the diet questionnaire
