# Supplementary material for: Weight-Loss Strategies Used by the General Population: How Are They Perceived?
Source: PLoS One. 2014 May 22;9(5):e97834. doi: 10.1371/journal.pone.0097834 (PMC4031181; doi:10.1371/journal.pone.0097834)
Supplement: Table S1 — Detailed information on the type of diet followed. (DOCX) [file pone.0097834.s002.docx]

Table S1 Detailed information on the type of diet followed

|  | Men | |  | Women | |  |
| --- | --- | --- | --- | --- | --- | --- |
|  | N | %total | % category | N | %total | % category |
| **Dietary recommendations** | **721** | **31.2** |  | **5311** | **29.4** |  |
| **Commercial diets** | **703** | **30.4** |  | **6152** | **34.1** |  |
| Dukan | 345 | 14.9 | 49.1 | 3031 | 16.8 | 49.3 |
| Consuming meal substitutes | 45 | 1.9 | 6.4 | 615 | 3.4 | 10.0 |
| Cohen | 49 | 2.1 | 7.0 | 595 | 3.3 | 9.7 |
| Chrononutrition | 50 | 2.2 | 7.1 | 301 | 1.7 | 4.9 |
| Cabbage soup | 53 | 2.3 | 7.5 | 250 | 1.4 | 4.1 |
| Montignac | 30 | 1.3 | 4.3 | 204 | 1.1 | 3.3 |
| Fricker | 5 | 0.2 | 0.7 | 96 | 0.5 | 1.6 |
| Dissociated | 14 | 0.6 | 2.0 | 85 | 0.5 | 1.4 |
| Scarsdale | 11 | 0.5 | 1.6 | 54 | 0.3 | 0.9 |
| Lemon detox | 4 | 0.2 | 0.6 | 40 | 0.2 | 0.7 |
| Raisin | 11 | 0.5 | 1.6 | 52 | 0.3 | 0.8 |
| Mayo | 7 | 0.3 | 1.0 | 25 | 0.1 | 0.4 |
| Low Carb | 4 | 0.2 | 0.6 | 16 | 0.1 | 0.3 |
| Atkins | 5 | 0.2 | 0.7 | 18 | 0.1 | 0.3 |
| Californian | 0 | 0.0 | 0.0 | 17 | 0.1 | 0.3 |
| Miami | 1 | 0.0 | 0.1 | 15 | 0.1 | 0.2 |
| The Zone | 1 | 0.0 | 0.1 | 5 | 0.0 | 0.1 |
| Ornish | 2 | 0.1 | 0.3 | 2 | 0.0 | 0.0 |
| Pinapple-Grapefruit | 0 | 0.0 | 0.0 | 5 | 0.0 | 0.1 |
| Hollywood | 1 | 0.0 | 0.1 | 3 | 0.0 | 0.0 |
| Other^1^ | 65 | 2.8 | 9.2 | 723 | 4.0 | 11.8 |
| **Commercial coaching programs** | **75** | **3.2** |  | **2280** | **12.6** |  |
| Weight Watchers | 73 | 3.2 | 97.3 | 2151 | 11.9 | 94.3 |
| Jenny Craig | 0 | 0.0 | 0.0 | 30 | 0.2 | 1.3 |
| Other^1^ | 2 | 0.1 | 2.7 | 99 | 0.5 | 4.3 |
| **Self-imposed dietary restrictions** | **759** | **32.8** |  | **3867** | **21.4** |  |
| Reducing quantity during meals | 343 | 14.8 | 45.2 | 1279 | 7.1 | 33.1 |
| Reducing snacking | 41 | 1.8 | 5.4 | 504 | 2.8 | 13.0 |
| Reducing fat intake | 47 | 2.0 | 6.2 | 172 | 1.0 | 4.4 |
| Reducing simple carbohydrates intake | 55 | 2.4 | 7.2 | 358 | 2.0 | 9.3 |
| Reducing fat and simple carbohydrates intake | 122 | 5.3 | 16.1 | 749 | 4.2 | 19.4 |
| reducing starchy food intake | 32 | 1.4 | 4.2 | 216 | 1.2 | 5.6 |
| Reducing fat and starchy food intake | 33 | 1.4 | 4.3 | 255 | 1.4 | 6.6 |
| Other^1^ | 86 | 3.7 | 11.3 | 334 | 1.9 | 8.6 |
| **Diets prescribed by a health professional** | **53** | **2.3** |  | **426** | **2.4** |  |

^1^Other diets were reported in plain text and later categorized according to the content in the main categories.
