# Supplementary material for: Weight-Loss Strategies Used by the General Population: How Are They Perceived?
Source: PLoS One. 2014 May 22;9(5):e97834. doi: 10.1371/journal.pone.0097834 (PMC4031181; doi:10.1371/journal.pone.0097834)
Supplement: Table S2 — Diet perception according to the type of diet followed – Cross-tabulations– Men (n = 2311). (DOCX) [file pone.0097834.s003.docx]

Table S2 Diet perception according to the type of diet followed – Cross-tabulations– Men (n=2311)

|  |  | **Adherence to dietary recommendations** | | **Commercial diet plan** | | **Self-imposed dietary restrictions** | | **Commercial coaching programs** | | **Diet prescribed by a health professional** | |  |
| --- | --- | --- | --- | --- | --- | --- | --- | --- | --- | --- | --- | --- |
|  |  | **N** | **%** | **N** | **%** | **N** | **%** | **N** | **%** | **N** | **%** | **P^a^** |
| Type of diet |  | 721 | 31.2 | 703 | 30.4 | 759 | 32.8 | 73 | 3.3 | 53 | 2.3 |  |
| ***Conditions of the diet*** |  |  |  |  |  |  |  |  |  |  |  |  |
| Reason for diet cessation | *Fixed duration/Objective attained* | 420 | 58.3 | 459 | 65.3 | 436 | 57.4 | 37 | 49.3 | 30 | 76.6 | 0.005 |
|  | *Early stop to the diet* | 301 | 41.8 | 244 | 34.7 | 323 | 42.6 | 38 | 50.7 | 23 | 43.4 |  |
| Diet duration | *< 1 month* | 375 | 52.0 | 461 | 65.6 | 476 | 62.7 | 38 | 50.7 | 23 | 43.4 | <.0001 |
|  | *≥ 1 month* | 346 | 48.0 | 242 | 34.4 | 283 | 37.3 | 37 | 49.3 | 30 | 48.0 |  |
| Concomitant physical activity | *Yes* | 418 | 58.0 | 270 | 38.4 | 348 | 48.9 | 32 | 42.7 | 31 | 48.0 | <.0001 |
|  | *No* | 303 | 42.0 | 433 | 61.6 | 411 | 54.2 | 43 | 57.3 | 22 | 42.0 |  |
| ***Perception of the diet*** | | | | | | | | | | | | |
| Adherence difficulty | *Very to quite easy* | 498 | 69.1 | 458 | 65.2 | 505 | 66.5 | 55 | 73.3 | 35 | 55.0 | 0.41 |
|  | *Moderately easy to very difficult* | 223 | 31.0 | 245 | 34.9 | 254 | 33.5 | 20 | 26.7 | 18 | 34.0 |  |
| Experiencing complications | *Not all to a little* | 582 | 80.7 | 459 | 65.3 | 628 | 82.7 | 49 | 65.3 | 37 | 69.8 | <.0001 |
|  | *Moderately to enormously* | 139 | 19.3 | 244 | 34.7 | 131 | 17.3 | 26 | 34.7 | 16 | 30.2 |  |
| Experiencing frustration | *Not all to a little* | 199 | 27.6 | 194 | 27.6 | 262 | 34.5 | 30 | 40.0 | 20 | 37.7 | 0.003 |
|  | *Moderately to enormously* | 522 | 72.4 | 509 | 72.4 | 497 | 65.5 | 45 | 60.0 | 33 | 62.3 |  |
| Hunger during dieting | *Not at all* | 318 | 44.1 | 369 | 52.5 | 309 | 40.7 | 39 | 52.0 | 29 | 54.7 | <.0001 |
|  | *A little to enormously* | 403 | 55.9 | 334 | 47.5 | 450 | 59.3 | 36 | 48.0 | 24 | 45.3 |  |

^a^P value obtained with Chi square tests
