# Supplementary material for: Weight-Loss Strategies Used by the General Population: How Are They Perceived?
Source: PLoS One. 2014 May 22;9(5):e97834. doi: 10.1371/journal.pone.0097834 (PMC4031181; doi:10.1371/journal.pone.0097834)
Supplement: Table S3 — Diet perception according to the type of diet performed – Cross-tabulations- Women (n = 18036). (DOCX) [file pone.0097834.s004.docx]

Table S3 Diet perception according to the type of diet performed – Cross-tabulations- Women (n=18036)

|  |  | **Adherence to dietary recommendations** | | **Commercial diet plan** | | **Self-imposed dietary restrictions** | | **Commercial coaching programs** | | **Diet prescribed by a health professional** | |  |
| --- | --- | --- | --- | --- | --- | --- | --- | --- | --- | --- | --- | --- |
|  |  | **N** | **%** | **N** | **%** | **N** | **%** | **N** | **%** | **N** | **%** | **P^a^** |
| Type of diet |  | 5311 | 29.5 | 6152 | 34.1 | 3867 | 21.4 | 2280 | 12.6 | 426 | 2.4 |  |
| ***Conditions of the diet*** | | | | | | | | | | | | |
| Reason for diet cessation | *Fixed duration/Objective attained* | 2514 | 47.4 | 2787 | 45.3 | 1860 | 48.1 | 844 | 37.0 | 181 | 42.5 | <.0001 |
|  | *Other* | 2795 | 52.7 | 3365 | 54.7 | 2006 | 51.9 | 1435 | 63.0 | 245 | 57.5 |  |
| Diet duration | *< 1 month* | 3148 | 59.3 | 4211 | 68.5 | 2663 | 68.9 | 1107 | 48.6 | 158 | 37.1 | <.0001 |
|  | *≥ 1 month* | 2162 | 40.7 | 1941 | 31.6 | 1203 | 31.1 | 1172 | 51.4 | 268 | 62.9 |  |
| Concomitant physical activity | *Yes* | 2720 | 51.2 | 2297 | 37.3 | 1639 | 42.4 | 1128 | 49.5 | 215 | 50.5 | <.0001 |
|  | *No* | 2590 | 48.8 | 3855 | 62.7 | 2227 | 57.6 | 1151 | 50.5 | 211 | 49.5 |  |
| ***Perception of the diet*** | | | | | | | | | | | | |
| Adherence difficulty | *Very to quite easy* | 3569 | 67.2 | 3292 | 53.5 | 2379 | 61.5 | 1632 | 71.6 | 289 | 67.8 | <.0001 |
|  | *Moderately easy to very difficult* | 1741 | 32.8 | 2860 | 46.5 | 1488 | 38.5 | 647 | 28.4 | 137 | 32.2 |  |
| Experiencing complications | *Not all to a little* | 3973 | 74.8 | 3472 | 46.4 | 2931 | 78.8 | 1540 | 67.6 | 308 | 72.3 | <.0001 |
|  | *Moderately to enormously* | 1337 | 25.2 | 2680 | 43.6 | 936 | 24.2 | 739 | 32.4 | 118 | 27.7 |  |
| Experiencing frustration | *Not all to a little* | 1419 | 26.7 | 1317 | 21.4 | 1032 | 26.7 | 949 | 41.6 | 149 | 35.0 | <.0001 |
|  | *Moderately to enormously* | 3891 | 73.3 | 4835 | 78.6 | 2835 | 73.3 | 1330 | 58.4 | 277 | 65.0 |  |
| Hunger during dieting | *Not at all* | 2353 | 44.3 | 3093 | 50.3 | 1532 | 39.6 | 1204 | 52.8 | 244 | 57.3 | <.0001 |
|  | *A little to enormously* | 2957 | 55.7 | 3059 | 49.7 | 2335 | 60.4 | 1075 | 47.2 | 182 | 42.7 |  |

^a^P value obtained with Chi-square tests
