# Supplementary material for: Weight-Loss Strategies Used by the General Population: How Are They Perceived?
Source: PLoS One. 2014 May 22;9(5):e97834. doi: 10.1371/journal.pone.0097834 (PMC4031181; doi:10.1371/journal.pone.0097834)
Supplement: Table S4 — Comparison between subjects having followed at least one weight loss diet in the past three years and subjects not having followed any; Analyses stratified by gender. (DOCX) [file pone.0097834.s005.docx]

Table S4 Comparison between subjects having followed at least one weight loss diet in the past three years and subjects not having followed any; Analyses stratified by gender

|  |  | Men (n=11 153) | | | |  | Women (n=36 317) | | | |  |
| --- | --- | --- | --- | --- | --- | --- | --- | --- | --- | --- | --- |
|  |  | Dieter | | Non dieter | | P-value | Dieter | | Non dieter | | P-value |
|  |  | N | % | N | % |  | N | % | N |  | % |
| N |  | 1 544 | 13.8 | 9 609 | 86.2 |  | 11 084 | 30.5 | 25 233 | 69.5 | <0.0001 |
| Age | ≤25 y-o | 26 | 1.7 | 235 | 2.5 | <0.0001 | 503 | 4.5 | 1 401 | 5.6 | <0.0001 |
|  | ]25-40] | 356 | 23.1 | 1 858 | 19.3 |  | 3 671 | 33.1 | 8 029 | 31.8 |  |
|  | ]40-60] | 624 | 40.4 | 3 226 | 33.6 |  | 4 982 | 45.0 | 10 263 | 40.7 |  |
|  | >60 | 538 | 34.8 | 4 290 | 44.7 |  | 1 928 | 17.4 | 5 540 | 22.0 |  |
| Educational level | No diploma and primary | 292 | 18.9 | 1 951 | 20.3 | 0.35 | 1 780 | 16.1 | 3 893 | 15.4 | 0.13 |
|  | Secondary | 295 | 19.1 | 1 873 | 19.5 |  | 2 305 | 20.8 | 5 137 | 20.4 |  |
|  | University | 957 | 62.0 | 5 785 | 60.2 |  | 6 999 | 63.2 | 16 203 | 64.2 |  |
| Current employement status | Work | 966 | 62.6 | 4 985 | 51.9 | <0,0001 | 7 314 | 66.0 | 15 579 | 61.7 | <0.0001 |
|  | Inactive | 578 | 37.4 | 4 624 | 48.1 |  | 3 770 | 34.0 | 9 654 | 38.3 |  |
| Smoking status | Never smoker | 558 | 36.1 | 4 019 | 41.8 | 0.0001 | 5 302 | 47.8 | 13 885 | 55.0 | 0.0001 |
|  | Former smoker | 765 | 50.0 | 4 337 | 45.1 |  | 4 150 | 37.4 | 7 598 | 30.1 |  |
|  | Current smoker | 221 | 14.3 | 1 253 | 13.0 |  | 1 632 | 14.7 | 3 750 | 14.9 |  |
| BMI | <25 | 505 | 32.7 | 5 683 | 59.1 | <0.0001 | 6 308 | 56.9 | 20 259 | 80.3 | <0.0001 |
|  | [25-30[ | 734 | 47.5 | 3 217 | 33.5 |  | 3 207 | 28.9 | 3 662 | 14.5 |  |
|  | ≥30 | 305 | 19.8 | 709 | 7.4 |  | 1 569 | 14.2 | 1 312 | 5.2 |  |
